# Rabies Virus Seroprevalence among Dogs in Limpopo National Park and the Phylogenetic Analyses of Rabies Viruses in Mozambique

**DOI:** 10.3390/pathogens11091043

**Published:** 2022-09-14

**Authors:** Milton Mapatse, Ernest Ngoepe, Darrell Abernethy, José Manuel Fafetine, Iolanda Anahory, Claude Sabeta

**Affiliations:** 1 Veterinary Faculty, University Eduardo Mondlane, Maputo 257, Mozambique; 2 World Organisation for Animal Health (WOAH) Rabies Reference Laboratory, Agricultural Research Council-Onderstepoort Veterinary Institute, Pretoria 0110, South Africa; 3 Centre for Veterinary Wildlife Research, University of Pretoria, Pretoria 0110, South Africa; 4 Department of Life Sciences, Aberystwyth School of Veterinary Science, Aberystwyth University, Aberystwyth SY23 3FL, UK; 5 Department of Diagnostics and Molecular Epidemiogy, Centre of Biotechnology (CB-UEM), University Eduardo Mondlane, Maputo 1100, Mozambique; 6 Central Veterinary Laboratory, Agricultural Research Institute of Mozambique, Maputo 1922, Mozambique; 7 Faculty of Veterinary Sciences, Department of Veterinary Tropical Diseases, University of Pretoria, Pretoria 0110, South Africa

**Keywords:** Limpopo National Park, Mozambique, dogs, rabies virus, rabies antibodies, phylogeny, seroprevalence

## Abstract

Rabies is considered a neglected disease among many developing Asian and African countries, including Mozambique, where its re-emergence is often attributed to low dog parenteral vaccination coverage. The objectives of this study were two-fold: (1) to assess the level of antibodies against rabies virus in dogs (n = 418) in Limpopo National Park (LNP), and (2) to genetically characterise selected rabies viruses from brain tissue samples collected in 2017 and 2018. To meet the first objective, we used the BioPro^TM^ Rabies blocking ELISA antibody kit, and the results were expressed as the percentage of blocking (%PB). Dog sera with PB ≥ 40% were considered positive for antibodies to rabies virus, whereas sera with PB < 40% were negative. Just under ninety percent (89.2%; n = 373) of dogs were seronegative, and the rest (10.8%; n = 45) had detectable levels of rabies virus-specific antibodies. All eight brain tissue samples were positive for rabies virus antigen using a direct fluorescent antibody test and amplified in a quantitative real-time PCR, but only five (n = 4 from dogs and n = 1 from a cat) were amplified in a conventional reverse-transcription PCR targeting partial regions of the nucleoprotein (N) and the glycoprotein (G) genes. All samples were successfully sequenced. Phylogenetically, the rabies viruses were all of dog origin and were very closely related to each other (Africa 1b rabies virus lineage). Furthermore, the sequences had a common progenitor with other rabies viruses from southern Africa, confirming the transboundary nature of rabies and the pivotal role of dogs in maintaining rabies cycles. The study demonstrates the principal application of the BioPro^TM^ rabies ELISA antibody for the detection of anti-lyssavirus-specific antibodies in the serum samples of dogs, and most importantly, it highlights the low levels of antibodies against rabies virus in this dog population.

## 1. Introduction

Rabies is an acute and fatal encephalitis caused by negative-sense RNA viruses of the *Lyssavirus* genus, order *Mononegavirales* and family *Rhabdoviridae* [1,2]. The prototype species, *Lyssavirus rabies*, is one of 17 currently recognised within the genus and is responsible for more than 99% of human rabies cases globally [1]. It is generally considered to be a transboundary disease that crosses national borders [3]. In Africa, approximately 21,000 people, representing 36% of all global rabies cases, are believed to succumb to dog-mediated rabies annually, although the true number is likely to be much higher [4,5].

In Mozambique, similar to other African countries, rabies is an endemic disease and a significant veterinary and public health problem that was first recognised in the early 1900s [6]. It is especially problematic in remote areas where diagnostic laboratory facilities and diagnostic capacity are generally lacking. Furthermore, a shortage of qualified personnel and insufficient medical and veterinary infrastructure compound the problem, leading to gross under-reporting of the disease [6,7,8].

Routine surveillance systems are largely inefficient, particularly in remote and resource-limited areas of the country, resulting in many patients seeking medical care outside of the established national health system [8,9,10]. In 2010, the first national rabies control strategy (2010–2014), proposed by the veterinary and health authorities [11], was approved. It aimed at reducing the incidence of dog bites and dog-mediated human rabies deaths through enhanced dog vaccination coverage and the establishment of more pre- and post-exposure prophylaxis centres throughout the country. These developments promoted responsible dog ownership and improved laboratory diagnostic capacity but did not yield the intended results, highlighted by the number of reported bite cases increasing from 15,222 in 2016 to 20,419 in 2017 [12]. In view of the low notification rates and an increase in dog bites and human rabies deaths, a second strategy was formulated and approved in 2019 [13]. This new strategy aimed to raise awareness among users of health centres. The focus was to improve rabies prevention measures; enhance vaccination coverage of dogs and cats; collect weekly epidemiological surveillance data; establish dog population control services in municipalities and local governments areas; identify dogs using microchips; and ensure pre-exposure prophylaxis for frontline rabies control and prevention professionals.

Between 2001 and 2017, dog rabies accounted for 79.5% (n = 898) of all clinically diagnosed animal rabies cases [14] in Mozambique. The rest were reported in cattle (7.9%), pigs (5.6%), domestic cats (4.9%), goats (1.8%), and a few (0.3%) in wild unspecified species [14]. In 2010, 9.2% (n = 123,910) of all dogs (n = 1,346,847) were vaccinated against rabies [15], while in 2018, the number declined to 8.2% (n = 247,321/3,011,656) [13], possibly due to poor awareness and inconsistent mass dog vaccination campaigns [12].

Despite there being four rabies diagnostic laboratories in Mozambique, there is a dearth of molecular epidemiological information about dog rabies in the country [8,16]. The Central Veterinary Laboratory is the main diagnostic laboratory, located in the capital city of Maputo. It is the only facility capable of diagnosing rabies using the gold standard direct fluorescent antibody test (DFAT) and reverse transcription-PCR (RT-PCR) [17]. In addition to conventional RT-PCR, qRT-PCR is recommended as a confirmatory diagnostic tool for rabies infection by both the World Organisation for Animal Health (WOAH) and the World Health Organization (WHO) [18,19,20]. However, simpler, less expensive diagnostic platforms are needed to enhance laboratory capacity in rabies endemic countries.

This investigation was undertaken to evaluate the level of serum antibodies to rabies virus infection among dogs in the Limpopo National Park (LNP) and to molecularly characterise rabies viruses (RABV), with the aim of assisting local veterinary and health authorities in their design of better rabies control programs.

## 2. Material and Methods

### 2.1. Study Area and Study Design

**Figure 1:**
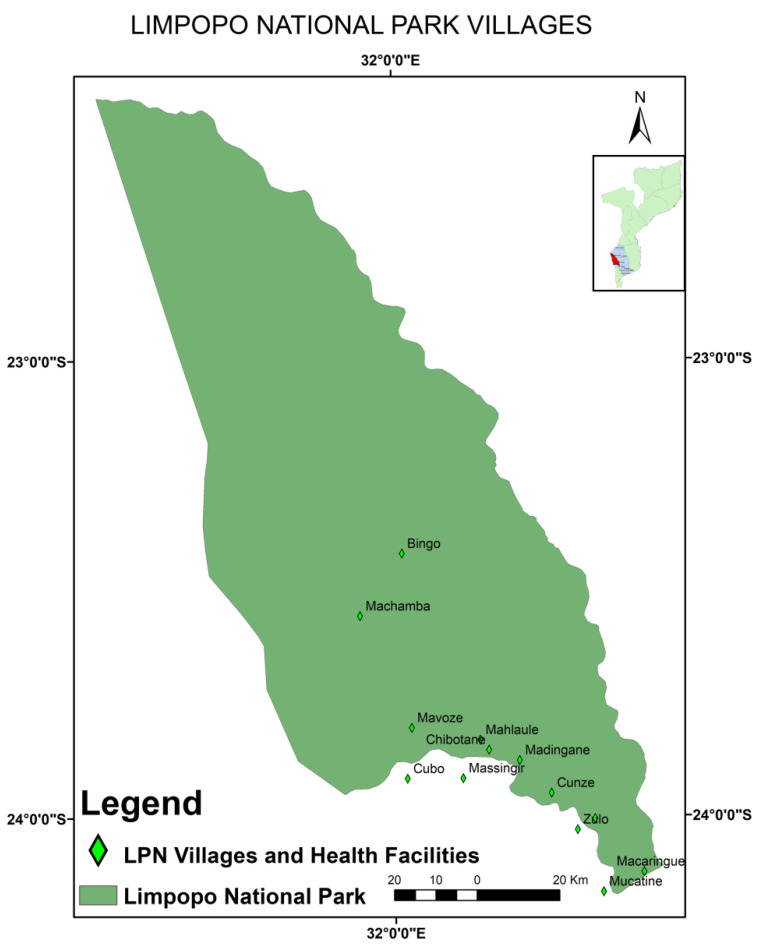
Limpopo National Park map, showing the study area [23].

To assess rabies seroprevalence, a cross-sectional study was carried out between November 2016 and April 2017 in Limpopo National Park (Figure 1), an area of approximately 11,000 km^2^, located to the west of Gaza Province and delimited by both the Limpopo (about 260 km) and Elefantes (about 85 km) rivers in the east and south, respectively [21,22].

### 2.2. Sampling and Source of the Samples

A sample size of 384 dogs was estimated for inclusion in the study, assuming 50% prevalence (as the real prevalence was unknown), a 5% margin of error, and 95% confidence intervals [24,25]. However, to increase precision, the sample size was increased to 418. The number of dogs per stratum (dogs per village) (Table S1) was calculated using the formula given by Cochran [26], taking into account the number of members in a household owning at least one dog. Blood samples were collected from the cephalic or saphenous vein using plain vacutainer blood tubes. The blood samples were centrifuged at 1050× *g* for 15 min (Centrifuge 5430, Eppendorf, Hamburg, Germany), then transferred to 2 mL sterile Eppendorf tubes, and stored at −40 °C until testing.

All owned dogs that were present in the owners’ homes or in the vicinity of nearby streets at the time of the visit were included, provided the owners verbally agreed to blood samples being taken. 

### 2.3. Viruses for Molecular Characterisation

Eight rabies virus-infected brain tissues collected between 2017 and 2018 were included in the study for molecular characterisation. These were confirmed infections in domestic dogs (*Canis familiaris*) (n = 7, from Maputo, Gaza, Sofala, and Nampula Provinces) and a domestic cat (*Felis catus*) (n = 1, from Sofala Province) using the DFAT in Maputo. Seven of the samples were provided by the Virology Section of the Central Veterinary Laboratory (CVL) in Maputo, and the remaining sample was obtained from a 6-month-old male dog of *Africanis* breed (LNP, Massingir district in Gaza Province) (Figure 1). The dog was euthanised after it presented typical signs of rabies virus infection.

### 2.4. Specimen Processing

#### 2.4.1. Blood Processing and ELISA

The blood samples were allowed to clot at 5 °C for two hours before the serum was decanted into 2 mL sterile Eppendorf tubes (Eppendorf, Hamburg, Germany). To ensure that a larger volume was obtained, the remaining serum was separated by centrifuging the blood samples at 615× *g* for 15 min (Centrifuge 5430^®^, Eppendorf, Hamburg, Germany), and immediately transferred to respective pre-labelled Eppendorf tubes. The serum samples were subsequently stored frozen at −40 °C until required for the serological analysis of antibodies to rabies virus using the BioPro^®^ Rabies ELISA antibody kit (O.K. Servis BioPro, Horni Pocernice, Czech Republic), according to the manufacturer’s guidelines.

Briefly, 60 µL of diluent buffer was distributed into dummy microplate wells, followed by 60 μL of serum samples or control sera. The sera and diluent were thoroughly mixed and 100 μL of diluted test and control sera were then dispensed into the rabies glycoprotein-coated microplates. 

The microplates were covered with adhesive foil and incubated overnight at 4 °C with gentle shaking at 125 RPM on an orbital shaker, and then washed six times. Thereafter, 100 µL of the biotinylated anti-rabies antibodies were distributed into each well and the plates were incubated for a further 30 min at 37 °C with gentle shaking. After incubation, four washes were performed before 100 µL of the streptavidin peroxidase conjugate was distributed into each well. This was followed by 30 min of incubation at 37 °C with gentle shaking, and a further four washes. Finally, 100 µL of 3,3′, 5,5-tetramethylbenzidine (TMB) chromogen solution was added to each well. The microplates were subsequently incubated in the dark for 20 min at room temperature with gentle shaking, and the reaction was stopped by the addition of 50 µL of a 0.5 M H_2_SO_4_ stop solution. 

The absorbance values were read at 450 nm using a microplate reader (Original Multiskan EX; Labsystems Inc., Helsinki, Finland). The percentages of the blocking values were calculated using a formula provided by the manufacturer. According to the manufacturer, dog sera with percentage blocking (PB) < 40% were considered negative for antibodies to rabies virus, whereas sera with PB ≥ 40% were considered positive.

#### 2.4.2. Lyssavirus Rabies Detection and Sequencing

RNA was extracted from the original RABV-infected brain tissues using TRI reagent (Sigma–Aldrich, St. Louis, MO, USA), as described in the manufacturer’s protocol. For both genes, the cDNA was synthetised, reverse transcribed, and amplified using the primers 001lys (+) 5′_1_ACGCTTAACGAMAAA_15_3′ and 550 (−) 5′_647_GTRCTCCARTTAGCRCACAT_666_3′ for a partial region of the nucleoprotein (N) gene and G_-_ (+) 5′_4665_GACTTGGGTCTCCCGAACTGGGG_4687_3′ and L (−) 5′_5543_CAAAGGAGAGTTGAGATTGTAGTC_5520_3′ [27,28] for a partial region of the glycoprotein (G) gene and the G-L intergenic regions of the rabies viruses. The annealing positions and polarity of the primers used were designated according to the Pasteur Virus (PV) genome [28].

The PCR amplicons for both partial regions of the N and G genes were purified from the reaction mixtures (comprising salts, nucleotides, primers, and primer dimers) using spin columns (Promega, Madison, WI, USA) and sequenced in both the forward and reverse directions with the same primers used in amplification reactions. Sequencing of the amplicons was performed on an ABI3700 sequencer using the Big Dye^TM^ v3.1 sequencing kit (Applied Biosystems, Massachusetts, Foster City, CA, USA), according to the manufacturer’s instructions.

Quantitative real time-PCR is a molecular method that allows for “real-time” monitoring and detection of amplified products in a PCR reaction. The assay in question was designed for the amplification and detection of a 126 bp fragment of the nucleoprotein gene of African lyssaviruses. It permits the rapid and accurate quantitative detection of African lyssavirus RNA and was performed following the protocol described by Coertse et al. [29].

#### 2.4.3. Phylogenetic Analysis

Phylogenetic analysis used an alignment of nucleotide sequences spanning the partial regions of the highly conserved N gene (647 bp) and the partial G gene and the G-L intergenic regions (518 bp) as inputs for tree reconstruction.

The partial N gene nucleotide sequences (n = 44) were aligned using the ClustalW subroutine of the MEGA X software package [30]. The best fitting nucleotide substitution model was identified as the transition model plus invariable sites and Gamma (TIM2 + I + G) using the Akaike information criterion (AIC) subroutine of the j-Model test software package (version 2.1.10). Similarly, 32 nucleotide sequences for the G-L intergenic region were aligned using ClustalW as before, and the best fitting nucleotide substitution model was found to be the transition model plus invariable sites (TIM1 + I). Phylogenetic analysis of both partial regions of the genome was performed using a Bayesian Markov Chain Monte Carlo (MCMC) method in the BEAST software package (version 2.5.0) using a relaxed exponential clock [31]. Three independent MCMC analyses sampled for 10 million states and a sampling frequency of 10,000 were combined after discarding at least 10% burn. The posterior distributions were subsequently inspected using Tracer software (version 1.7.1) to ensure adequate mixing and convergence before the associated statistics were summarised as a maximum clade credibility tree and visualised using FigTree v 1.4.4.

### 2.5. Statistical Analysis

The statistical analysis was focused on calculating the frequency of the values of interest. Binary logistic regression was used to determine the associations between the level of rabies antibodies and the independent variables (sex and age of the dogs). Ninety-five percent confidence intervals (CI 95.0%) and the *p* values were calculated using SPSS Statistics software for Windows version 18.0 (SPSS Inc., Chicago, IL, USA).

## 3. Results

### 3.1. Seroprevalence of Rabies Virus

All of the dogs included in this study were of the *Africanis* breed. Specific details of the dog population and their vaccination status and antibody levels against rabies virus are shown in Table 1. 

The majority (n = 280, 66.9%) of surveyed dogs were male, 39.2% were less than one year of age, 15.6% were one to two years of age, and the remaining 45.2% (n = 189) were over two years of age. Nearly 20% (n = 83) had a history of vaccination or a rabies certificate confirming vaccination at least once in the year prior to sampling. Most of the dogs (n = 335, 80.1%) had no vaccination history or rabies certificate. Across both vaccinated and unvaccinated groups, only 10.8% (n = 45) of the dogs were seroconverted. 

Table 2 shows the frequency of vaccinated and unvaccinated dogs according to the age and sex groups, and serological status according to the dog vaccination records. Younger dogs were in the lower percentage for both the vaccinated and non-vaccinated dogs (*p* > 0.05), while males had higher proportions for both statuses (*p* < 0.05). Of the total number of unvaccinated dogs (n = 335), only 8.7% had antibodies to rabies virus and of these, 1.5% (n = 5) were under 1 year of age.

The proportion of seronegative dogs was significantly greater than the proportion of seropositive dogs, and there was a direct association between seropositivity and increasing age (*p* < 0.05) (Table 3). The results presented in Table 4 reveal that all (100%; n = 8) brain samples submitted to DFAT were positive for rabies virus.

### 3.2. Phylogenetic Analysis

**Figure 2:**
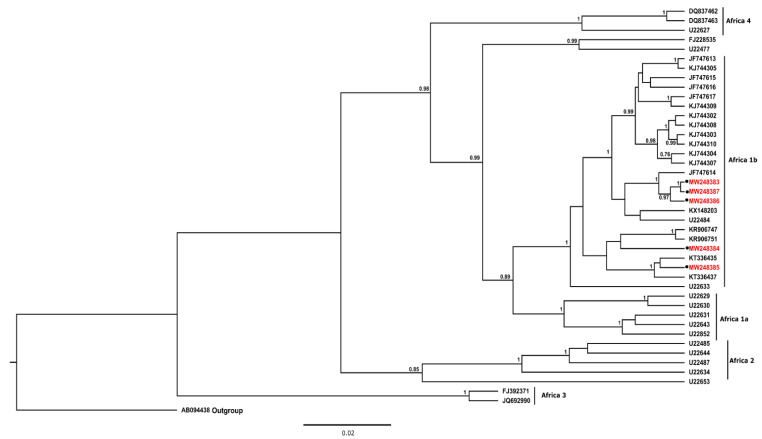
Maximum clade credibility tree illustrating the genetic relationships among rabies viruses from Mozambique and neighbouring countries. Nucleotide sequences of a partial region of the N gene were used in the analysis. The phylogenetic analysis was conducted using BEAST software version 2.5.0. [31] with posterior probabilities of 0.75. The posterior probabilities are shown on nodes supporting the branches and only those equal to or above 0.75 were retained. The analysis involved 44 rabies nucleotide sequences, and those used in this study (Table S2) are represented by a dark circle and red letters.

**Figure 3:**
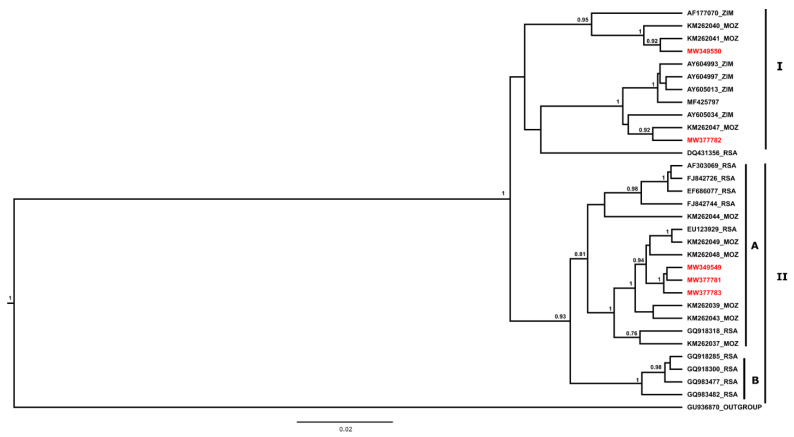
Maximum clade credibility tree showing the placement of the five Mozambican RABVs. Nucleotide sequences of a partial region of the glycoprotein gene and the G-L intergenic region were used in the analysis. The phylogenetic analysis was conducted using BEAST software version 2.5.0. [31] with posterior probabilities of 0.75. The posterior probabilities are shown on the nodes supporting the branches, and only those equal to or above 0.75 were retained. The analysis involved 32 nucleotide sequences, and those used in this study (Table S3) are represented by red letters.

For the N gene, phylogenetic analysis demonstrated that all lyssaviruses recovered from dogs and a cat from Mozambique were closely related and clustered with other rabies viruses of dog origin from South Africa. The rabies viruses shared approximately 99% nucleotide sequence homology and 96% nucleotide sequence identity with those from neighbouring Zimbabwe and Tanzania. All rabies viruses included in the study belonged to the Africa 1b RABV lineage (Figure 2). For the G gene, the rabies viruses from Nampula (MW349550) and a feline rabies virus from Sofala (MW377782) clustered with previously characterised rabies viruses from Mozambique and Zimbabwe within Clade I (Figure 3). The remaining rabies viruses (MW349549, MW377781, and MW377783) clustered with rabies viruses from other host species from Mozambique and South Africa in clade II-A (Figure 3).

## 4. Discussion and Conclusions

The immunization of dogs remains one of the most cost-effective measures for controlling rabies. It has the potential to reduce dog-mediated human rabies cases by drastically reducing the number of dogs susceptible to infection [32], as demonstrated in North America and Europe [33]. In these regions, other measures, including the removal of stray dogs and responsible dog ownership (i.e. movement restriction), have been pivotal towards the elimination of dog rabies [34]. Latin America provides a more recent example of how parenteral dog vaccination has contributed to the elimination of dog-mediated human rabies [5,35,36]. In Mozambique, the low seroprevalence of rabies virus among dogs elevates the risk for the possible spillover of RABV infection into wildlife carnivore species, humans, and companion animals within the LNP and national parks in neighbouring countries [37,38,39].

The low number of seroconverted dogs detected in our investigation and in a study by Simone [40] in Manica Province, Mozambique highlights the risk of contracting RABV in the event of an outbreak. To our knowledge, this is the first study investigating the seroprevalence against RABV infection in a conservation area in Mozambique. While we utilised antibody tests, Moore et al. [41] stated that population-level antibody prevalence in dogs is not a perfect means of assessing the effectiveness of oral rabies vaccine baits. Indeed, antibodies are not the only informative measure of immunity against RABV infection. Both cellular and humoral immunity, as well as innate immunity, are crucial in preventing disease. Observations from a challenge study on several animal species demonstrated that animals with antibody levels above the 40% PB threshold associated with the BioPro ELISA were, in fact, equipped with better humoral responses to vaccination and infection [41].

The findings obtained from the serology experiments of our study underscore the need for local governments and related authorities to invest more in the parenteral immunization of dogs. 

In 2015, about a year prior to our study commencing, a small-scale, anti-rabies vaccination campaign was conducted in the region. Vaccination coverages ≥ 70% of the canine population are needed to break the transmission cycles between dogs and humans [13,41]. Many factors contribute to low vaccination coverage in LNP, resulting in inadequate antibody titres among dogs in this remote region. These include but are not limited to inconsistent parenteral vaccination campaigns; a lack of information among communities about impending vaccination campaigns; the absence of owners at the time of vaccination; a lack of awareness about the need to vaccinate dogs; and an apparent lack of time for household owners to take their pets for vaccination [23].

Although 8.7% of unvaccinated dogs (29/335) had detectable anti-rabies antibody titres, the likelihood of false positive reactions was low [42]. This ELISA is unlikely to be affected by non-specific reactions compared to virus-neutralising antibody assays, such as RFFIT and FAVNT. Neutralising antibody tests use live viruses and cells, and thus, they are very sensitive to non-specific reactions, resulting in false positives [43]. Moore et al. [41] showed a stronger correlation between the RFFIT and the indirect ELISA (from Biorad) values, but it was not linear, since the two assays measure different antibody functions. In our study, dog vaccination history was provided not only by the heads of households but also by teenage children and may be susceptible to recall bias. Antibody levels to rabies virus detected in unvaccinated dogs may also have been derived from maternal antibodies rather than direct infection [44], or from anamnestic immune responses to previous natural rabies exposures [45,46]. The latter has been also reported in studies carried out in Nigeria, Kenya, and India [42,44,47,48], and additional research is warranted to verify these findings.

Interestingly, most of the dogs < 1 year of age in our study had no vaccination history. We, therefore, advocate that veterinary authorities consider immunization campaigns for younger dogs and also carefully consider including puppies at six weeks of age in accordance with the recommendations proposed by Arega et al. [49]. This is especially important since puppies do not have appreciable levels of maternally-derived antibodies. Furthermore, maternally-derived antibodies do not limit the protective efficacy of inactivated adjuvanted rabies vaccines [49]. Younger dogs are also easier to capture and handle for parenteral vaccination than free-roaming adults who may benefit from baited vaccines such as Raboral-VRG. In addition, in India, it was demonstrated that humans are susceptible to RABV-infection from puppies less than three months old [50].

As shown in other similar studies [51,52], and irrespective of vaccination status, the adult dogs in our study had significantly higher levels of anti-rabies-specific antibodies than younger dogs. It is thus likely that they have received more than one vaccination. In a study carried out to understand the ecology of dogs in LNP by Mapatse [53], 70.2% of respondents reported that their dogs were given to them as a gift, implying that they may have been previously vaccinated. There was no statistically significant association between gender and rabies antibody titres (*p* > 0.05) in dogs, agreeing with the findings from other independent studies [54,55,56].

Phylogenetic analysis, based on partial regions of the N and G genes, showed that the rabies viruses (RABVs) recovered from domestic animals all belonged to the Africa 1b RABV lineage. These viruses, in addition to being closely related, clustered with RABVs from Tanzania, Zimbabwe, RSA, and Mozambique [57], suggesting a common progenitor [58] and confirming the transboundary nature of rabies. The Africa 1b RABV variants, which include the canid variant of southern Africa [42,44], also clustered together. The genetic relationship between the RABV lineages in this study strengthens the evidence for the historical introduction of rabies to the sub-region and Mozambique in the late 1950s [59]. Furthermore, the data demonstrate that rabies viruses currently circulating in the country are very closely related, considering that the Mozambican viruses are grouped (in clades I and II) with those from the neighbouring countries (along the borders of Zimbabwe and South Africa).

In Mozambique, the diagnosis of most animal rabies cases is based on clinical signs and the history of bites rather than laboratory confirmation [8,17]. Very few brain samples are submitted from the field (in distant provinces) to diagnostic laboratories [60]. In cases where samples are submitted from distant locations, they reach the diagnostic facility in a decomposed state and are consequently not suitable for the DFAT. Passive surveillance data for most canine rabies endemic countries, including Mozambique, are inadequate for estimating the burden of the disease. Therefore, after thorough validation, molecular tests should be considered as alternatives and applied to samples that test negative using DFAT but where there was a known human contact/bite. Traditional techniques, such as Seller’s staining, have now been discontinued [18] and are no longer recommended by the WOAH. Other immunohistochemical tests, such as the dRIT, which has similar diagnostic sensitivity and specificity as the gold standard DFAT, are therefore appropriate as field-based assays and can increase the number of field samples tested, thereby improving rabies surveillance. 

The canid RABV variant is maintained primarily in dog populations in Mpumalanga and Limpopo Provinces on the eastern seaboard, with occasional spillover into wildlife host species, including the black-backed jackal (*Canis mesomelas*) and bat-eared fox (*Otocyon megalotis*) in South Africa [61], and more recently, the aardwolf [62]. These three wildlife hosts maintain RABV independently from domestic dogs [63,64]. It is, therefore, important to understand the rabies transmission dynamics between domestic and wildlife hosts in Mozambique in order to break the rabies transmission cycles.

The findings from our study demonstrate that the dog vaccination campaigns in Mozambique are not consistent. Hence, vaccination coverage and educating communities about the public health hazards of rabies and responsible dog ownership should be substantially enhanced. Rabies surveillance and laboratory diagnosis are still inadequate and there is a clear need for field technicians and veterinarians to submit more samples to diagnostic laboratories. The decentralisation of rabies testing and the introduction of point-of-care diagnostics is another option that may overcome low sample submission for rabies surveillance in Mozambique. Such measures can overcome the current problem of underreporting of rabies, not only in Mozambique but throughout the whole region. Rabies viruses identified in dogs in the current study are similar to the rabies virus variant circulating in dogs in the neighbouring countries of South Africa and Zimbabwe. This observation not only highlights the important and pivotal role that domestic dogs play in rabies transmission cycles in Mozambique but also the transboundary nature of the disease. 

The data from this study will serve to strengthen rabies control programs and interventions in Mozambique through the formulation of appropriate and targeted vaccination campaign strategies, particularly in hot spot areas in rural communities, including LNP. The veterinary and public health service sectors in the Massingir District should ensure that epidemiological surveillance systems are more effective by reviewing the policies and strategies for disease control, as the level of protection for dogs against rabies virus infection is currently ineffective.

## 5. Study Limitations

The lack of accurate, systematic, and up-to-date dog population census data was the main limiting factor, as it made it extremely difficult to ascertain vaccination coverages. Other limiting factors included a lack of research data pertaining to animal rabies cases in Mozambique and a low number of samples submitted for diagnosis in the central and regional laboratories of the country. From 2017 to 2018, only eight brain samples from dogs and a cat from the five provinces of the country were submitted to the CVL, the main laboratory of the Ministry of Agriculture and Rural Development.

## Figures and Tables

**Table 1:** Details of the dog population and their vaccination and rabies virus seroprevalence status.

Variables	Frequency (%)
Dog age groups	
<1 Year	164 (39.2)
1–2 Years	65 (15.6)
>2 Years	189 (45.2)
Sex of the dogs	
Male	280 (66.9)
Female	138 (33.1)
History of dog vaccination	
Unvaccinated	335 (80.1)
Vaccinated	83 (19.9)
Antibody Level	
PB < 40% (Negative)	373 (89.2)
PB ≥ 40% (Positive)	45 (10.8)

**Table 2:** Serological status and proportion of vaccinated and unvaccinated dogs according to age and sex.

Variables	Frequency (%)		
Age of dogs	Unvaccinated	Vaccinated	Total
<1 Year	141 (42.1)	22 (26.5)	163
1–2 Years	54 (16.1)	12 (14.5)	66
>2 Years	140 (41.8)	49 (59.1)	189
Sex of dogs	Unvaccinated	Vaccinated	Total
Male	216 (77.1)	64 (22.9)	280
Female	119 (86.2)	19 (13.8)	138
Vaccination status according to PB	Negative	Positive	Total
Unvaccinated	306 (91.3)	29 (8.7)	335
Vaccinated	67 (80.7)	16 (19.3)	83

**Table 3:** Serological results according to age and sex of dogs.

Variables	Frequency (%)		Total	*p*-Value
	Negative	Positive		
**Group age**				
<1 Year	156 (95.7)	7 (4.3)	163 (100)	0.001
1–2 Years	61 (92.4)	5 (7.6)	66 (100)	0.001
>2 Years	156 (82.5)	33 (17.5)	189 (100)	0.059
Total	373 (89.2)	45 (10.8)	418 (100)	
**Sex**				
Female	124 (89.9)	14 (10.1)	138 (100)	0.774
Male	249 (88.9)	31(11.1)	280 (100)	
Total	373 (89.2)	18 (10.8)	418 (100)	

**Table 4:** Results of rabies virus antigen detection using DFAT and PCR.

Lab Reference	Animal	Collection Site	DFAT	RT-PCR	qRT-PCR	Gene Copies/µL
496/18	Canine	Maputo	Positive	Negative	Positive	640
597/18	Canine	Sofala	Positive	Negative	Positive	789
501/18	Canine	Gaza	Positive	Negative	Positive	708
MW248383	Canine	Gaza	Positive	Positive	Positive	6.4 × 10^6^
124/18	Canine	Maputo	Positive	Positive	Positive	1 × 10^7^
393/18	Feline	Sofala	Positive	Positive	Positive	2.5 × 10^6^
468/17	Canine	Gaza	Positive	Positive	Positive	8.8 × 10^6^
368/18	Canine	Nampula	Positive	Positive	Positive	1 × 10^7^

## Data Availability

All relevant data are within the manuscript and its Supplementary Materials.

## Supplementary Materials

The following supporting information can be downloaded at: https://www.mdpi.com/article/10.3390/pathogens11091043/s1, Table S1: Details of the sample size of the canine population covered in this study; Table S2: Rabies virus sequences used for partial N gene phylogenetic analysis; Table S3: Rabies virus sequences used for G gene phylogenetic analysis.
