# Supplementary material for: Rabies Virus Seroprevalence among Dogs in Limpopo National Park and the Phylogenetic Analyses of Rabies Viruses in Mozambique
Source: Pathogens. 2022 Sep 14;11(9):1043. doi: 10.3390/pathogens11091043 (PMC9506193; doi:10.3390/pathogens11091043)
Supplement: Supplementary file 1 [file pathogens-11-01043-s001.zip › pathogens-1766405-supplementary.pdf]

Table S1. Details of the sample size of the canine population covered in this study.

Village	Total number of dogs per selected village*	Intended sample size per stratum	Sample size retrieved per stratum
Bingo	63	37	64
Macaringue	126	74	51
Munhamane	56	33	44
Madingane	81	47	51
Malhaule	57	33	33
Machamba	64	37	75
Cunze	63	37	52
Mavoze	147	86	48
Total	657	384	418

*** Source: [65]**

Table S2. Rabies virus sequences used for partial N gene phylogenetic analysis

Virus#	Host Species of origin	Year of Collection	Country of origin	Lineage	Genbank Accession Number
343/18	Canine	2018	Mozambique	Africa 1b	MW248383
368/18	Canine	2018	Mozambique	Africa 1b	MW248384
393/18	Feline	2018	Mozambique	Africa 1b	MW248385
468/17	Canine	2017	Mozambique	Africa 1b	MW248386
124/18	Canine	2018	Mozambique	Africa 1b	MW248387
UPV130	Canine	2008	RSA	Africa 1b	JF747614
10_509	Canine	2010	RSA	Africa 1b	KJ744304
UPV150	Canine	2007	RSA	Africa 1b	JF747615
10_274	Canine	2010	RSA	Africa 1b	KJ744308
10_387	Canine	2010	RSA	Africa 1b	KJ744303
11_300	Canine	2011	RSA	Africa 1b	KJ744307
10_268	Canine	2010	RSA	Africa 1b	KJ744302
10_458	Caprine	2010	RSA	Africa 1b	KJ744309
UPV153	Canine	2007	RSA	Africa 1b	JF747616
11_217	Canine	2011	RSA	Africa 1b	KJ744310
8721AFS	Human	2008	RSA	Africa 1b	U22633
UPV128	Canine	2008	RSA	Africa 1b	JF747613
UPV167	Canine	2007	RSA	Africa 1b	JF747617
11_185	Canine	2011	RSA	Africa 1b	KJ744305
9137ALG	Canine	1982	Algeria	Africa 1a	U22643
87012MAR	Canine	1986	Morocco	Africa 1a	U22631
9107MAR	Human	1990	Morocco	Africa 1a	U22852
8693GAB	Canine	1986	Gabon	Africa 1a	U22629
8698GAB	Canine	1986	Gabon	Africa 1a	U22630
86031MOZ	Mouse	1986	Mozambique	Africa 1b	KX148203
8631MOZ	Canine	1986	Mozambique	Africa 1b	U22484
RV2775.1	Canine	2010	Tanzania	Africa 1b	KR906747
RV2780.1	Canine	2011	Tanzania	Africa 1b	KR906751

34312	Canine	2012	RSA	Africa 1b	KT336437
21467	Canine	1993	Zimbabwe	Africa 1b	KT336435
8697BEN	Canine	1995	Benin	Africa 2	U22485
8660GUI	Canine	1986	Guinea	Africa 2	U22487
9218TCH	Canine	1992	Chad	Africa 2	U22644
8801CAM	Canine	1987	Cameroon	Africa 2	U22634
8692EGY	Human	1979	Egypt	Africa 4	U22627
32/02	Yellow Mongoose	2002	RSA	Africa 3	FJ392371
57-06	Yellow Mongoose	2006	RSA	Africa 3	JQ692990
dog/S2/1999	Canine	1999	Egypt	Africa 4	DQ837462
dog/S3/1999	Canine	1999	Egypt	Africa 4	DQ837463
9126MEX	Canine	1991	Mexico	America 1	U22477
DgNYKprwsky1950	Canine	1950	USA	America	FJ228535
8738THA	Human	1983	Thailand	Asia	U22653
SPU94.06	Human	2006	RSA	outgroup	DQ676932
Aravan	Myotis blythii	2002	Kyrgyzstan	outgroup	AB094438

Table S3. Rabies virus sequences used for G gene phylogenetic analysis.

Virus #	Host Species	Year of Collection	Country/Province	Genbank Accession Number
124/18	Canine	2018	Mozambique (Maputo)	MW349549
368/18	Canine	2018	Mozambique (Nampula)	MW349550
343/18	Canine	2018	Mozambique (Gaza)	MW377781
393/18	Feline	2018	Mozambique (Sofala)	MW377782
468/17	Canine	2017	Mozambique (Gaza)	MW377783
22547	Canine	1994	Zimbabwe	AF177070
332	Canine	1997	RSA	AF303069
29103	Canine	2003	Mutare, Zimbabwe	AY604993
29406	Feline	2003	Glendale, Zimbabwe	AY604997
29175	Human	2003	Rusape, Zimbabwe	AY605013
28460	Canine	2002	Mutoko, Zimbabwe	AY605034
204/14	Canine	2014	Zimbabwe	MF425797
35/00	Canine	2000	RSA	EF686077
333/06	Canine	2006	RSA	EU123929
652/08	Canine	2008	RSA	FJ842726
206/07	Canine	2007	RSA	FJ842744
108/00	Canine	2000	RSA	GQ918285
132/92	Canine	1992	RSA	GQ918300
151/86	Canine	1986	RSA	GQ918318
326/86	Canine	1986	RSA	GQ983472
341/88	Canine	1988	RSA	GQ983477
366/84	Canine	1984	RSA	GQ983482

298.93	Canine	1993	Mozambique	KM262037
572.99	Canine	1999	Mozambique	KM262039
633.00	Canine	2000	Mozambique	KM262040
315.04	Canine	2004	Mozambique	KM262041
558.05	Canine	2004	Mozambique	KM262043
659.05	Canine	2005	Mozambique	KM262044
482.12	Canine	2012	Mozambique	KM262047
1018.12	Bovine	2012	Mozambique	KM262048
233.13	Canine	2013	Mozambique	KM262049
RV131	Bat	2010	Zimbabwe	GU936870

Reference

65. Serviços Distritais de Actividades Económicas de Massingir. *Censo Efectivos Animais Pecuários Massingir 2015*; Report No.: N/A. SDAE, Massingir, Mozambique, 2015.
